# Axenic interspecies and intraclonal hybrid formation in *Leishmania*: Successful crossings between visceral and cutaneous strains

**DOI:** 10.1371/journal.pntd.0010170

**Published:** 2022-02-09

**Authors:** Camino Gutiérrez-Corbo, Bárbara Domínguez-Asenjo, Yolanda Pérez-Pertejo, Carlos García-Estrada, Felio J Bello, Rafael Balaña-Fouce, Rosa M. Reguera

**Affiliations:** 1 Departamento de Ciencias Biomédicas, Facultad de Veterinaria, Universidad de León, León, Spain; 2 Facultad de Ciencias Agropecuarias, Programa de Medicina Veterinaria, Universidad de la Salle, Bogotá, Colombia; University of Washington, UNITED STATES

## Abstract

Diseases caused by trypanosomatids are serious public health concerns in low-income endemic countries. Leishmaniasis is presented in two main clinical forms, visceral leishmaniasis—caused by *L*. *infantum* and *L*. *donovani*—and cutaneous leishmaniasis—caused by many species, including *L*. *major*, *L*. *tropica* and *L*. *braziliensis*. As for certain other trypanosomatids, sexual reproduction has been confirmed in these parasites, and formation of hybrids can contribute to virulence, drug resistance or adaptation to the host immune system. In the present work, the capability of intraclonal and interspecies genetic exchange has been investigated using three parental strains: *L*. *donovani*, *L*. *tropica* and *L*. *major*, which have been engineered to express different fluorescent proteins and antibiotic resistance markers in order to facilitate the phenotypic selection of hybrid parasites after mating events. Stationary and exponential-phase promastigotes of each species were used, in *in vitro experiments*, some of them containing LULO cells (an embryonic cell line derived from *Lutzomyia longipalpis*). Several intraclonal hybrids were obtained with *L*. *tropica* as crossing progenitor, but not with *L*. *donovani* or *L*. *major*. In interspecies crossings, three *L*. *donovani* x *L*. *major* hybrids and two *L*. *donovani* x *L*. *tropica* hybrids were isolated, thereby demonstrating the feasibility to obtain *in vitro* hybrids of parental lines causing different tropism of leishmaniasis. Ploidy analysis revealed an increase in DNA content in all hybrids compared to the parental strains, and nuclear analysis showed that interspecies hybrids are complete hybrids, i.e. each of them showing at least one chromosomal set from each parental. Regarding kDNA inheritance, discrepancies were observed between maxi and minicircle heritage. Finally, phenotypic studies showed either intermediate phenotypes in terms of growth profiles, or a decreased *in vitro* infection capacity compared to the parental cells. To the best of our knowledge, this is the first time that *in vitro* interspecies outcrossing has been demonstrated between *Leishmania* species with different tropism, thus contributing to shed light on the mechanisms underlying sexual reproduction in these parasites.

## Author summary

Neglected Tropical Diseases (NTDs) represent a serious threat to humans, especially for those living in poor or developing countries. Leishmanianiosis is considered a zoonotic NTD transmitted by the bite of female phlebotomine sandflies, and is manifested mainly as a visceral form (caused by *L*. *infantum* and *L*. *donovani*) and a cutaneous form (caused by many species including *L*. *major*, *L*. *tropica* and *L*. *braziliensis*). Although it is now known that sexual reproduction occurs in these parasites, more studies are necessary to characterize the ability of *Leishmania* to generate hybrids, which may represent an important mechanism for virulence, drug resistance or adaptation to the host immune system. Therefore, several experiments were conducted to generate either intraclonal or interspecies hybrids *in vitro*. Results demonstrated that hybrids can be formed even with outcrosses between parasites causing visceral and cutaneous forms of the disease. Characterization of hybrids in terms of ploidy, kDNA content, growth rate and infection capacity provide important information about sexual reproduction in these parasites.

## Introduction

Major pathogenic trypanosomatids, *Trypanosoma brucei* (responsible for sleeping sickness in Africa), *Trypanosoma cruzi* (responsible for Chagas disease in South America) and *Leishmania* spp. are single-celled protists causing some of the most neglected and deadly transmissible diseases produced by eukaryotes in low-income countries. *Leishmania* is a digenetic parasite transmitted to humans by species of sandflies of the genus *Phlebotomus* and *Lutzomyia*, which maintain the zoonotic cycle [1,2]. In the human host, *Leishmania* causes different disorders: i) cutaneous leishmaniasis, produced among other species by *L*. *major* and *L*. *tropica* in the Old World and by *L*. *braziliensis* in the New World, is the less severe form and may lead to the formation of disfiguring sores and scars in exposed parts of the body [3]; ii) mucocutaneous leishmaniasis is a more severe presentation that can destroy the mucous membranes of mouth and nose [4]; iii) visceral leishmaniasis is responsible for hepato-splenomegaly and can be fatal if left untreated. It is produced by *L*. *donovani* and *L*. *infantum* in the Old World, and by *L*. *infantum chagasi* in the New World, and can derive into a rare skin form of the disease called post-kala azar dermal leishmaniasis when the antimony therapy fails [5,6].

The mode of reproduction of these species is no longer just a basic biology issue, but a source of information that can provide explanations to important clues regarding parasite virulence, drug resistance or adaptation to the host immune system. Nowadays, it is unquestionable that sexual reproduction and related processes such as gene recombination and chromosome segregation, takes place in trypanosomatids. As for other eukaryotes, meiosis-specific genes for the formation of gametes by reductive division during meiosis as well as their cytoplasmic fusion (syngamy) during mating, have been described in trypanosomatids [7–9]. The debate is now focused on determining how frequent is sex and what is the impact exerted on species and natural populations of these parasites from an evolutionary point of view [10–15].

Since the late 80s, there is confirmed evidence that, under controlled laboratory conditions, different strains of *T*. *brucei* are capable of generating viable hybrids by genetic exchange [16]. It was observed that filial hybrid generations inherited gene markers from each parent strain. In addition, this genetic exchange took place in the salivary glands of tsetse flies [16,17]. The filial generation shared characteristics from both parental strains (biparental inheritance), but some specific characteristics such as the occurrence of a proportion of filial hybrids with variable ploidy [18], or the existence of mating types capable of self- and non-self-recognition, were also reported [19]. Finally, thanks to the use of transgenic fluorescent strains, mating phenomena and gamete production could be observed [20–22].

The results obtained with *Leishmania* spp. are more recent and confirm those obtained with other trypanosomatids. The use of antibiotic resistance markers served to detect the first interclonal genetic exchange event between different strains of *L*. *major* in the presence of either the natural vector *Phlebotomus duboscqi*, or the permissive *Lutzomyia longipalpis* sandfly. Similar to *T*. *brucei*, a percentage of the hybrid generation had an unexpectedly higher ploidy than that of the parental strains [23,24]. Furthermore, viable hybrids that partly retained the visceralization capacity in mice were obtained between two identical clones of *L*. *infantum* (self-mating or selfing) [25]. Moreover, hybrids were also produced between strains of different species, such as *L*. *infantum* and *L*. *major*, whose filial hybrid generation retained part of the characteristics of each parent [26].

Until 2020, genetic exchange events in *Leishmania* had only been demonstrated in the midgut of the permissive *Lu*. *Longipalpis* or non-permissive *Ph*. *papatasi* and *Ph*. *duboscqi* sand flies fed with infected blood. However, a recent work demonstrated that these events could also take place between promastigotes in the absence of the insect vector in axenic conditions [27], and might be possible even between amastigotes within the macrophage phagolysosome [28]. These new findings could significantly facilitate this type of study.

Other important results obtained from genetic exchange processes were related to the inheritance of extrachromosomal kinetoplast DNA (kDNA). kDNA is an intricate mesh of both maxicircles (few in number and equivalent to mitochondrial DNA) and minicircles (very numerous and responsible for the editing of parasite mRNAs) [29]. Genetic exchange experiments with different strains of *T*. *brucei* showed that unlike minicircles [30], the DNA content of the maxicircles from filial hybrids seems to have uniparental origin, although recent results suggest the early biparental inheritance of maxicircles [31].

Encouraged by the importance of genetic exchange in *Leishmania* we have proceeded to perform interspecies and intraclonal hybridization studies between lines of *L*. *donovani*, *L*. *tropica* and *L*. *major*. These experiments have been conducted in the absence of the vector, but assessing the effect of an embryonic cell line from the permissive vector *Lu*. *longipalpis* (LULO cells) as feed layer for hybridization experiments. The inheritance of the hybrids obtained from each of the parental strains, their ploidy, kDNA composition, viability and infectivity in macrophages have been analysed to contribute to decipher the mechanism behind reproduction in *Leishmania*.

## Materials and methods

### Ethics statement

Experiments in this study involving animals have been carried out in accordance with Spanish and European Union legislation (RD 53/2013 and 2010/63/EU, respectively). The protocols used in this study have been approved by the Ethics Committee of the University of León (Spain), Project license number OEBA-ULE-007-2019.

### Animals and infections

Six-to-eight week old female Balb/c mice, purchased from Janvier Laboratories (St Berthevin Cedex, France) were used to maintain the *Leishmania* strains. Mice were infected intraperitoneally with 5x10^8^ metacyclic promastigotes of different *Leishmania* strains (see below) and after 6 weeks, animals were euthanized. Livers, spleens and lymph nodes were aseptically processed in order to recover the parasites as previously described [32,33].

### Parasites and growth curves

Parental strains, *L*. *donovani* MHOM/ET/67/HU3, also known as LV9 or L-82 (kindly provided by Dr. Philippe Loiseau, Université Paris-Sud, Paris, France), *L*. *major* LV39c5 (RHO/SU/59/P) (kindly provided by Dr. Stephen M Beverley, Washington University School of Medicine, Saint Louis, USA), *L*. *tropica* MRAT/IQ/72/ADHANIS1 (kindly provided by Dr. José M. Requena, CBM, UAM, Madrid, Spain) and hybrid parasites were cultured at 26°C in M199 medium (Sigma), supplemented with 25 mM HEPES pH 6.9, 10 mM glutamine, 7.6 mM hemin, 0.1 mM adenosine, 0.01 mM folic acid, 1x RPMI 1640 vitamin mix (Sigma), 10% (v/v) heat-inactivated fetal bovine serum (FBS), and antibiotic mixture (200 U/mL penicillin and 200 μg/mL streptomycin). Stationary-phase parasites were harvested after 3 days in this phase, while exponential-phase promastigotes were collected 3 days after starting the culture. Both exponential and stationary phase promastigotes were confirmed microscopically before performing the experiments.

To prepare growth curves, parental and hybrid lines were seeded at a density of 10^6^ promastigotes/mL in complete M199 medium and parasite numbers were determined daily on a Z1 Beckman Coulter.

### LULO cell cultures

LULO cells were kindly provided by Dr. Felio Jesús Bello (Universidad de la Salle, Sede Norte, Bogotá, Colombia). LULO is an adherent embryonic cell line derived from the permissive sandfly *Lutzomyia longipalpis* [34]. LULO cells were cultured in monolayer at 26°C in L-15:Grace´s (1:1) medium (both from FisherScientific) supplemented with 10% FBS and antibiotic mixture until reaching confluence between passages. Interaction between parental promastigotes and LULO cells was tested by seeding 2x10^4^ LULO cells in 8-well Ibidi chambers and adding promastigotes 24 hours later at a 1:5 ratio. Interaction was visualized by confocal microscopy. For this purpose, parasites were stained with Hoechst 33342 (Fisher Scientific) during 30 minutes at 37°C and images were acquired on a Zeiss LSM800 confocal microscope.

### Generation of transgenic lines of *Leishmania* expressing fluorescent marker genes

To perform *in vitro* crossing experiments, one of the transgenic lines was generated previously in our lab, namely *L*. *major* mCh HYG (expressing the gene encoding mCherry (mCh) and the hygromicin resistance cassette (HYG)). In addition to this transgenic line, some new fluorescent cell lines were generated after electroporation of the corresponding plasmids (see below) and further selection of the parasites on semisolid medium containing specific antibiotic markers (see below) [35].

On the one hand, *L*. *donovani* mCh HYG and *L*. *tropica* mCh HYG were obtained after electroporation of these strains with pLEXSY-mCh-HYG construct [33] and subsequent selection of transfectants in medium containing hygromycin (200 μg/mL). On the other hand, electroporation of promastigotes of *L*. *tropica* with pLEXSY-iRFP-PAC [32] and further selection of transfectants in medium with puromycin (200 μg/mL), gave rise to *L*. *tropica* iRFP PAC, which constitutively expresses the *iRFP* gene. Finally, electroporation of promastigotes of *L*. *donovani*, *L*. *major* and *L*. *tropica* with the construct pLEXSY-CTN-PAC (see below) and selection of transfectants in medium with puromycin (200 μg/mL) gave rise to *L*. *donovani* CTN PAC, *L*. *major* CTN PAC and *L*. *tropica* CTN PAC, respectively, which produce the citrine (CTN) protein. For the construct of pLEXSY-CTN-PAC (S1 Fig), the gene encoding CTN was amplified by PCR from the plasmid pLEXSY-CTN-HYG [25] using the primers RBF921, which contains the sequences for XhoI and BglII at the 5´-end, and RBF614, the latter containing the sequences for NotI at the 5´-end (Table 1). The fragment obtained (CTN; 720 bp) included the kozak sequence for improved expression of the fluorescence protein. Then, it was digested with XhoI and NotI, and cloned in a pBluescript SK (Agilent), thus forming pSK-CTN fragment. In the next step, plasmids pSK-CTN and pLEXSY-hyg2 (Jena Bioscience) were digested with BglII and NotI, and the fragment CTN was cloned in the pLEXSY-hyg2 vector, thus obtaining pLEXSY-hyg2-CTN. Finally, pLEXSY-CTN-PAC was generated after digestion of pLEXSY-hyg2-CTN with SpeI and NotI in order to replace the fragment HYG-utr2 with the fragment PAC-HSP70. This fragment was obtained from pLEXSY-iRFP-70-PAC after digestion with SpeI and NotI, and contains the HSP70 downstream region, which has been reported to help increase the expression of the reporter gene included in pLEXSY vectors [32].

**Table 1:** Primers used in this work.

Product name	Primers	Product size (bp)	Use
Citrine [25]	RBF921, FW: *ccgCTCGAGgaAGATCTCCACC*ATGGTGAGCAAGGGCGAGG RBF614, RV: *ataagaatGCGGCCGC*TTACTTGTACAGCTCGTCCATG	720	ORF cloning
Hygromycine [33]	RBF646, FW: ATGAAAAAGCCTGAACTCACC RBF647, RV: CTATTCCTTTGCCCTCGGAC	1025	Characterization of transformants
Puromycin [32]	RBF774, FW: ATGACCGAGTACAAGCCCACG RBF775, RV: TCAGGCACCGGGCTTGCGG	720	Characterization of transformants
mCherry	TOX43, FW: TGGCCATCATCAAGGAGTTCA TOX44, RV: CCCTCGGCGCGTTCGT	711	Characterization of transformants
Citrine	TOX41, FW: CGAGGAGCTGTACACCGGG TOX42, RV: ACGAACTCCAGCAGGACCAT	717	Characterization of transformants
iRFP [32]	RBF843, FW: ATGGCGGAAGGATCCGTCGC RBF844, RV: TCACTCTTCCATCACGCCGATC	950	Characterization of transformants
Calmodulin-binding	TOX59, FW: CTGAACCGGATGCAGATTCGTGAG TOX60, RV: CTTGTTGCGTGTCGTCTCGATCTG	786	Genomic SNP-CAPs
ABC transporter	TOX61, FW: ATGGACTTGGTGCAGCTGCAGCGA TOX62, RV: GATGTCATCGCACAGGGACGTAATG	2091	Genomic SNP-CAPs
Sec20	TOX63, FW: GGACCAGGCGCTACACGAGCTGAT TOX64, RV: GACACGCGCAGCAGCAGATCATCG	618	Genomic SNP-CAPs
Rad9	TOX65, FW: GGCTGAGCGACGGCATCGAGATCAG TOX66, RV: CCTCGTTGGCGGAGGTGTAGGTGC	1956–1959	Genomic SNP-CAPs
Asparaginase	TOX69, FW: CGTCGCCGGATACCTGACGGAGC TOX70, RV: GGTGATTTCGCCTCGCAGATTCTG	1015	Genomic SNP-CAPs
Paraflagellar rod protein	TOX67, FW: ATGAGCATCGCTGCGGACATGGCGT TOX68, RV: CTACTCGGTGATCTGTCGCACCGTC	1800	Genomic SNP-CAPs
Ribosomal protein L22p/L17e	TOX55, FW: ATGCCGAAGCCAGCCCCTCGGTAC TOX56, RV: CAGCAGTGATGGCGGGCACGTCCA	829	Genomic SNP-CAPs
Fumarate hydratase (Fumerase)	TOX45, FW: ATGTCTCTGTGCGACCA TOX46, RV: TCACGCAAGCGTCTTCG	1707	Genomic SNP-CAPs
Aquaglyceroporin 1	TOX51, FW: GGCTACGCGAGTATGTTGCC TOX52, RV: AAGACATACAAGAACATGCCGA	688–736	Genomic SNP-CAPs
2-oxoisovalerate dehydrogenase beta subunit	TOX49, FW: AGCGTTCAACCGAGTGAGTT TOX50, RV: AAGTGCACCACGGACTTGAT	1025	Genomic SNP-CAPs
A2 [36]	L2: TTGGCAATGCGAGCGTCACAGTC R3: CAACGCGTACGATAATGCCACA	230/480	Genomic amplification
ACTIN	TOX83, FW: AATGGCTGACAACGAGCAGA TOX84, RV: CACTTGTTGTGCACGATGCT	1125	Genomic sequencing
ITS [37]	(LITSR); TOX75, FW: CTGGATCATTTTCCGATG (L5.8S); TOX76, RV: TGATACCACTTATCGCACTT	420	Strain confirmation
ND7 [38]	TOX97, FW: GTGCATTTATGCGTTTATTAATGTG TOX98, RV: ACAACATCAACATTACCAATAACTGC	800	Maxicircle SNP-CAPs
CYTB [26]	TOX99, FW: AGCGGAGAGRARAGAAAAGG TOX100, RV:: GYTCRCAATAAAATGCAAATC	618	Maxicircle SNP-CAPs
ND5 [26]	TOX103, FW: GAYGCDATGGAAGGACCDAT TOX104, RV: CCACAYAAAAAYCAYAANGAACA	456	Maxicircle SNP-CAPs
12sRNA [26]	TOX101, FW: AACTARTGAWGGCACAGTTGTTCT TOX102, RV: ACCCAACTAACGAATTGCWTTT	818	Maxicircle SNP-CAPs
MINICIRCLES 1 (MIN 1) [39]	TOX73, FW: CCAGTTTCCCGCCCCG (KDNA-F) TOX74, RV: GGGGTTGGTGGTGTAAAATAG (KDNA-R)	780	Minicircle amplification
MINICIRCLES 2 (MIN 2) [40]	TOX75, FW: TAATATAGTGGGCCGCGCAC TOX76, RV: CCGACATGCCTCTGGGTAGG	<250–1000	Minicircle amplification

The correct integration of both the reporter and antibiotic resistance cassettes in the *Leishmania* genome was tested by PCR. In addition, fluorescent signal was checked by flow cytometry (MACSQuant Analyzer 10) and confocal microscopy. For this purpose, 2x10^4^ exponential phase promastigotes were collected, washed twice in 1xPBS and placed in 8-well Ibidi chambers previously treated with poly-L-lysine (Sigma). Parasites were stained with Hoechst 33342 (Fisher Scientific) during 30 minutes at 37°C and images were acquired on a Zeiss LSM800 confocal microscope.

### Crossing experiments

*In vitro* hybrids were generated by mixing equal numbers (2.5x10^6^) of parental promastigotes (exponential or stationary-phase promastigotes), either on empty 96 well plates or on 96 well plates previously seeded with confluent LULO cells. Several days after mixing parental cell lines (3, 6 or 9 days, depending on the experiment) parasites were transferred into 48-well plates including fresh complete M199 medium containing the selection antibiotics (puromycin and hygromycin, 100 μg/mL each), in order to select the double resistant hybrids generated due to genetic exchange.

### Analysis of DNA content

Ploidy of hybrids and parental strains was determined through the analysis of their total DNA content. Briefly, 1x10^6^ promastigotes were collected, washed twice in 1xPBS and fixed by adding 70% ethanol dropwise in vortex. Samples were kept at -20°C during at least 24 hours and up to 1 week until analysis. For this, fixed cells were washed again in 1xPBS twice, stained with propidium iodide (40 μg/mL) and treated with 200 μg/mL RNase (Sigma) at 37°C for 30 minutes. Analysis was carried out on MACSQuant Analyzer 10 flow cytometry equipment. Data were analysed with FlowJo v10 software. *L*. *major* Friedlin (largely diploid) and *L*. *braziliensis* Mb2904 (approximately 3n) were used as ploidy-content reference cell lines in order to compare them with hybrids and parental strains.

### Identification of parental *Leishmania* strains

The characterization of parental *Leishmania* strains was performed by sequencing ITS1 (Internal Transcribed Spacer 1 region between the *ssu* and 5–8 rRNA genes)[37], using the primers described in Table 1.

### Single Nucleotide Polymorphisms Cleaved Amplification Polymorphic site (SNP-CAPs)

Analysis of specific loci placed on different chromosomes and kinetoplastid DNA in the interspecific hybrids (*L*. *donovani* mCH HYG x *L*. *major* CTN PAC and *L*. *donovani* mCh HYG x *L*. *tropica* iRFP PAC) and in their parental strains was carried out by SNP-CAPs using the SNP2CAPS tool described by Thiel et al., (2004) [41]. Detailed description of the genes, primers, SNPs, restriction enzymes used for this purpose and the size of the predicted products in each *Leishmania* strain can be found in Table 2.

**Table 2:** Genes tested by SNP-CAPs. The gene name, gene identification (ID), location, the name of forward and reverse primers used for PCR amplification (sequence of these primers can be found in Table 1), SNP position, as well as the expected band sizes after digestion with the suitable restriction enzymes, are shown.

Gene name	ID	Location	Primers	SNP position	RE	Predicted products (bp)		
						*L*. *donovani*	*L*. *major*	*L*. *tropica*
Calmodulin-binding	LdBPK.01.2.000260 LMJLV39_010007500 LTRL590_010007500	Chr. 1	TOX59 TOX60	608	MscI (MlsI)	608 + 178	786	786
ABC Transporter	LdBPK.03.2.000150 LMJLV39_030006600 LTRL590_030006400	Chr. 3	TOX61 TOX62	206	NruI	2091	1885 + 206	n.a
					PmlI	2091	n.a	1812 + 279
Sec20	LdBPK.11.2.000300 LMJLV39_110008400 LTRL590_110008700	Chr. 11	TOX63 TOX64	158 + 204	BcnI (NciI)	414 + 158 + 46	618	618
Rad9	LdBPK.15.2.001040 LMJLV39_150016800 LTRL590_150016300	Chr. 15	TOX65 TOX66	938	MluCI (TasI)	1018 + 938	1956	n.a
				491	AvaII (Eco47I)	1956	n.a	1468 + 491
cytoplasmic l-asparaginase i-like protein	LdBPK.15.2.000440 LMJLV39_150009800 LTRL590_150009200	Chr. 15	TOX69 TOX70	832	EcoRV (Eco32I)	832 + 183	1015	1015
Paraflagellar rod protein	LdBPK.16.2.001510 LMJLV39_160021000 LTRL590_160021500	Chr. 16	TOX67 TOX68	925	XhoI	1800	925 + 875	n.a
				1441	NruI	1441 + 359	n.a	1800
Ribosomal protein L22p/L17e	LdBPK.22.2.000700 LMJLV39_220013900 LTRL590_220012400	Chr. 22	TOX55 TOX56	593	MluCI (TasI)	593 + 236	829	829
Fumarate hidratase	LdBPK.29.2.002080 LMJLV39_290027300 LTRL590_290026600	Chr. 29	TOX45 TOX46	935	MluCI (TasI)	1707	955 + 772	955 + 772
Aquaglyceroporin 1	LdBPK.31.2.000030 LMJLV39_310005100 MG797692.1	Chr. 31	TOX51 TOX52	279	MluCI (TasI)	457 + 279	736	688
2-oxoisovalerate dehydrogenase beta subunit	Ld .35.2.209420 LMJLV39_350005400 LTRL590_350005500	Chr. 35	TOX49 TOX50	609	HpaI (KspAI)	609 + 416	1025	1025
CYTB	FJ416603.1 MK514113.1 MN904525.1	Maxicircle	TOX99 TOX100	618	TatI	420 + 140 + 60	252 + 228 + 141	n.a.
					NdeI	621	n.a.	525 + 96
ND5	FJ416603.1 MK514113.1 MN904525.1	Maxicircle	TOX103 TOX104	456	BglII	464	258 + 206	n.a.
					EcoRI	335 + 129	n.a.	464

### Determination of inheritance in polyploid hybrids

In order to determine the origin of the extra chromosome sets in the different hybrids, we based on the experiment carried out by Romano et al in 2014 [26]. Simulated 3n and 4n hybrids were created by mixing parental DNA in 1:1, 1:2 and 2:1 ratios (for the *L*. *donovani* x *L*. *major* hybrids) and 1:1, 1:3 and 3:1 ratios (for the *L*. *donovani* x *L*. *tropica* hybrids). Then, several loci were analysed by SNP-CAPs as indicated above. Subsequently, the ratio obtained between the largest band digested with the restriction enzyme belonging to one parental allele, and the uncut band corresponding to the allele of the other parental, was calculated using the Gene Tools 4.3.9 (Syngene) software. The ratio obtained in simulated 3n and 4n was compared to that obtained by the hybrids, thus allowing us to classify the hybrids according to one of the control ratios by statistical analysis using k-means analysis in SPSS COR statistical software.

### *In vitro* infections

The ability of intraclonal and interspecies hybrids to infect *in vitro* was tested in RAW murine macrophages. For this purpose, 5x10^4^ RAW cells were seeded in 8-well Ibidi chambers and infected in a proportion of 1:10 with promastigotes. After 2 hours, the co-culture was washed three times in PBS. Three days after the infection, samples were stained with Hoechst 33342 (Fisher Scientific) during 30 minutes at 37°C, and images were acquired on a Zeiss LSM800 confocal microscope.

The *in vitro* infective competence of hybrids *vs*. parents was determined using bone marrow-derived macrophages (BMMs) obtained from Balb/c mice. Briefly, after euthanasia, the animals were dissected and the femur and tibia were cleaned and separated in sterile conditions using scissors and forceps. The heads of the bones were cut off, and 5 mL of 1xHBSS (Hanks Balanced Salt Solution) were injected through the bone channel with a 25G syringe. The undifferentiated cells were collected [42] and frozen. When needed, cells were thawed and the protocol proposed by Legarda was followed [43]. Briefly, cells were placed in 5 mL of differentiation medium, which contains RPMI supplemented with 10% FBS and 30% L929 supernatant (L929 fibroblast cells produce rM-CSF produce rM-CSF [recombinant macrophage colony stimulating factor]) in a small petri dish to allow them to attach, and 24 hours later, they were collected and seeded in 10 mL of differentiation medium in a 10-cm non-treated petri dish. On day 4, 5 mL of differentiation medium were added and BMMs were ready for parasite infection on day 7. Amastigotes of each parental and hybrid strains were added to differentiated-macrophages at 1:10 ratio for 6 hours and maintained at 37°C. Later, non-internalised parasites were washed twice with PBS. After 3 days, samples were fixed in 100% methanol and stained with Giemsa. Then, pictures were acquired with a Nikon microscope and 100 macrophages per parasite-cell-line were analysed for the presence of amastigotes, which were counted in three independent experiments.

## Results and discussion

### Generation of engineered parental cell lines

**Fig 1:**
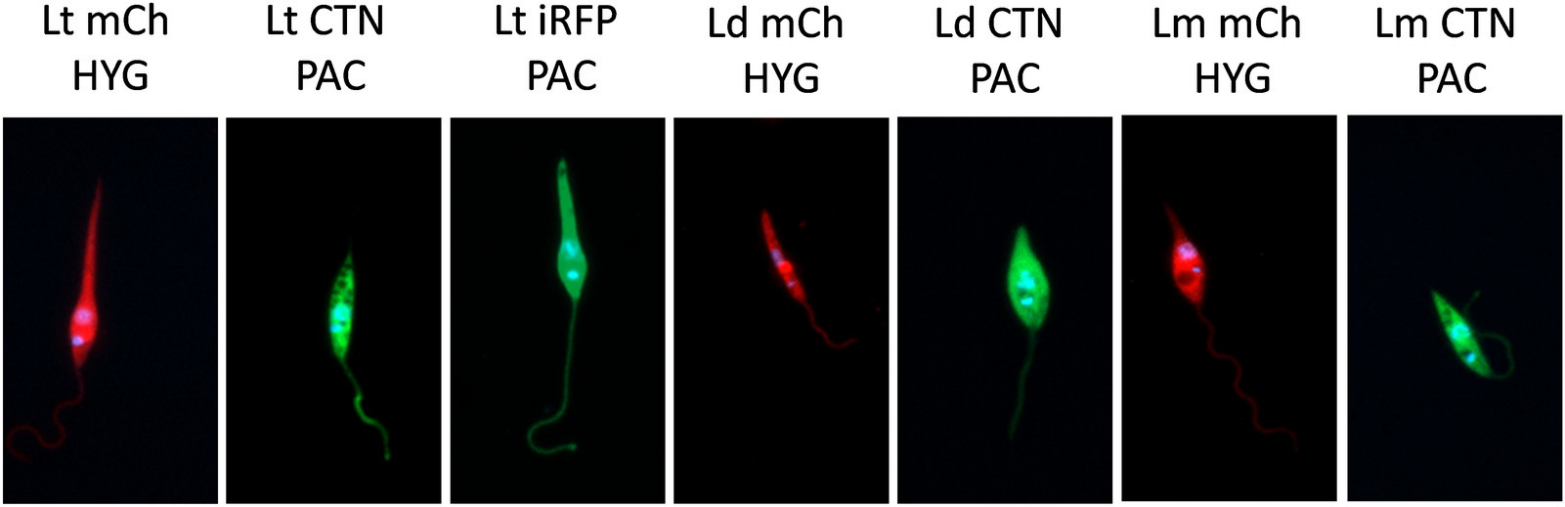
Engineered *Leishmania* parental strains. Representative confocal microscopy pictures of promastigotes of the parental fluorescent strains of *Leishmania* used for the intraclonal and interspecies outcrossings. Lt mCh HYG: *L*. *tropica* mCh HYG (561 nm); Lt CTN PAC: *L*. *tropica* CTN PAC (488 nm); Lt iRFP PAC: *L*. *tropica* iRFP PAC (640 nm); Ld mCh HYG: *L*. *donovani* mCh HYG (561 nm); Ld CTN PAC: *L*. *donovani* CTN PAC (488 nm); Lm mCh HYG: *L*. *major* mCh HYG (561 nm); Lm CTN PAC: *L*. *major* CTN PAC (488 nm). The wavelength refers to the excitation laser.

In order to ease the phenotypic analysis of the parental and hybrid strains, in addition to *L*. *major* mCh HYG, which had been already generated in our laboratory and was able to stably express the gene encoding mCh and the hygromycin resistance cassette [33], we prepared six other novel transgenic strains (see Materials and methods). After the analysis of these transgenic strains by PCR to confirm the integration of both the reporter and antibiotic resistance cassettes, and analysis by flow cytometry (MACSQuant Analyzer 10), the fluorescent parasites were visualized with different excitation wavelengths by confocal microscopy (Fig 1).

### Formation and characterization of intraclonal hybrid strains *in vitro*

**Fig 2:**
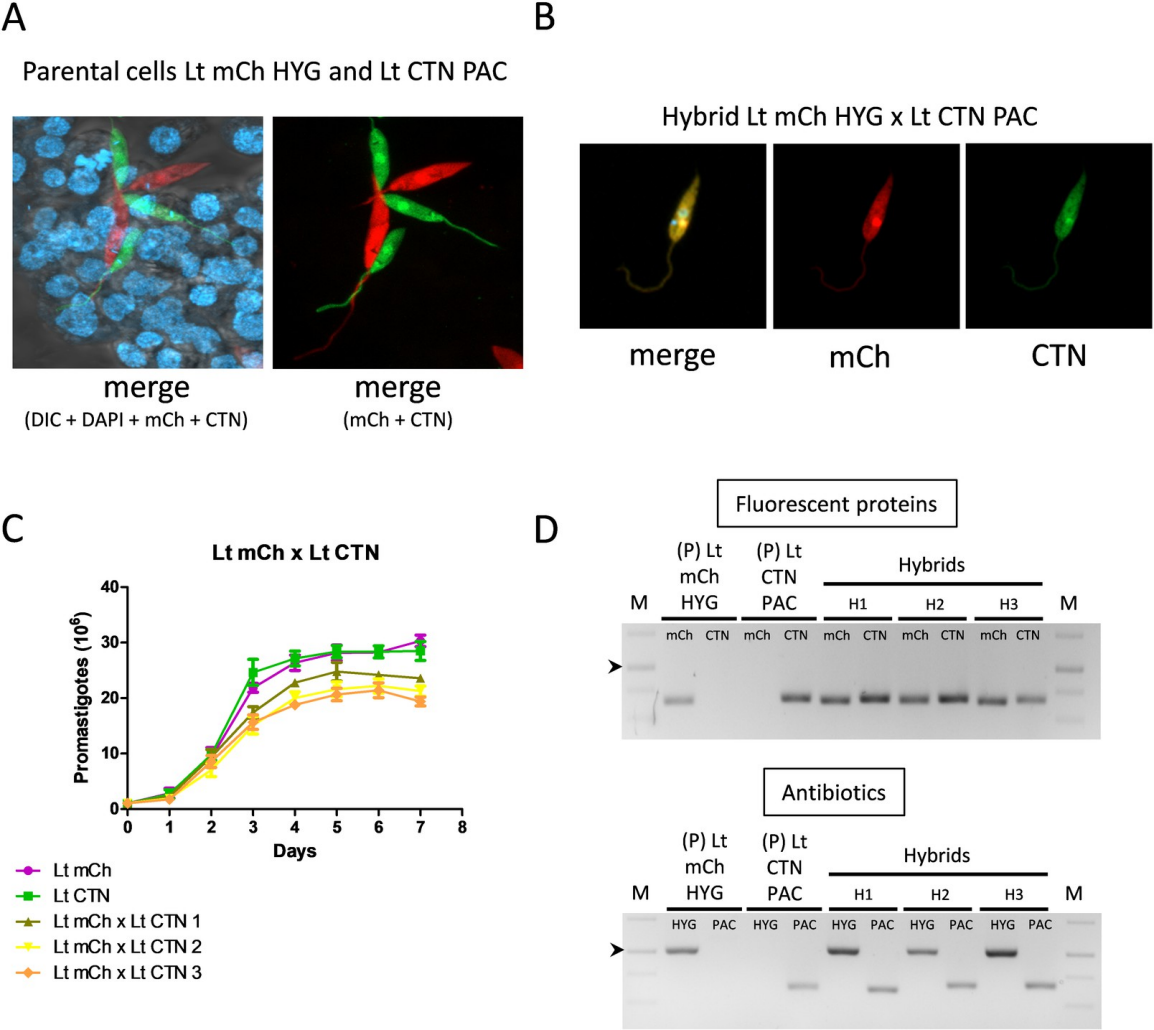
Characterization of intraclonal hybrids. (A) Confocal microscopy showing the interaction between LULO cells and parental cell lines of *L*. *tropica* before mating. (B) Representative image of confocal microscopy of one hybrid clon of *L*. *tropica* mCh HYG x *L*. *tropica* CTN PAC and fluorescence emission in the mCh (561 nm) and CTN (488 nm) channels. (C) Growth curves (number of promastigotes in culture during 7 days) of the parental *L*. *tropica* mCh HYG (Lt mCh) and *L*. *tropica* CTN PAC (Lt CTN), and three representative intraclonal hybrids (Lt mCh x Lt CTN 1, 2 and 3). (D) Agarose gels of the electrophoresis of the PCR products obtained after the amplification of the genes encoding fluorescent proteins (mCh and CTN) and antibiotic resistance (HYG or PAC) in the parental (P) and three representative intraclonal hybrids (H). The arrowheads indicate the 1000 bp band in the DNA molecular weight marker (M).

Stationary-phase promastigotes of *L*. *tropica* mCh HYG and *L*. *tropica* CTN PAC were mixed in the presence of a feed layer of cells isolated from *Lu*. *longipalpis* embryos (LULO cells) (Fig 2A), and the double selection of antibiotics (puromycin and hygromycin) was started at three different time points: days 3, 6 and 9 post-mixing. Two different experiments were carried out, and as shown in Table 3, the highest ratio of hybrid formation was achieved when antibiotic selection was performed at day 3 post-mixing, which could be related to direct competition between the newly-formed hybrid and the parental promastigotes. If hybrids are formed relatively soon after parental contact, and antibiotic selection is added at late times (9 days), development of the newly-formed hybrid could be impaired regarding parental cells due to biological competition mechanisms, thus avoiding the recovery of hybrids at late time. However, if selection is performed soon after hybrid formation, competition with parental strains would be lower, since the latter would die, thereby promoting the growth of hybrids bearing antibiotic resistance cassettes in the genome.

**Table 3:** Intraclonal hybrid formation in *L*. *tropica*

	Addition of antibiotics (days post-mixing)	Percentage of positive wells
*L*. *tropica* mCh HYG x *L*. *tropica* CTN PAC	3 days	Exp. 1: 2.34% (9/384)
		Exp. 2: 10.67% (41/384)
	6 days	Exp. 1: 1.3% (5/384)
		Exp. 2: 2.6% (10/384)
	9 days	Exp. 1: 0.78% (3/384)
		Exp. 2: 0% (0/384)

These hybrids have yellow color (using both red and green channels for microscopic visualization) (Fig 2B), are resistant to puromycin and hygromycin, and show lower growth rate (lower number of promastigotes during the stationary phase) than the parental lines (Fig 2C). To confirm the inheritance of DNA from parental strains in the hybrid filial strains, we amplified the genes conferring antibiotic resistance and the genes encoding fluorescent proteins using the primers listed in Table 1. Fig 2D shows agarose gels of the amplification products of the *L*. *tropica* strain before mating and in three hybrids. These results clearly show that unlike the parental strains, the hybrids contain genes from both parents either for antibiotic resistance or for fluorescence emission, thus confirming that genetic exchange events occurred during the outcrossing.

Typically, the outcrossing experiments carried out in trypanosomatids have been performed in the midgut of the vector’s digestive tract between promastigotes [23–26,44,45]. These experiments consisted of feeding the insect vectors with blood infected with both strains of parental promastigotes to be crossed, with mating taking place in the midgut of the insect (in the case of *Leishmania*). A recent paper, however, described the mating of two different strains of *L*. *tropica* promastigotes in axenic conditions [27]. The yield of hybrids obtained, although significant, was much lower than that obtained in the presence of the insect [45]. Considering these results, we performed intraclonal mating experiments *in vitro* using a feed layer of LULO cells, which were used to mimic the conditions within the insect midgut. *L*. *tropica* was initially chosen as model microorganism due to the fact that this species has a greater mating success in flies [45]. Stationary-phase promastigotes were selected because they are less mobile and allowed us to maintain the culture for up to 9 days without disturbing the LULO cell monolayer.

We also attempted to generate intraclonal hybrids for other *Leishmania* species, namely *L*. *major* (*L*. *major* mCh HYG x *L*. *major* CTN PAC) and *L*. *donovani* (*L*. *donovani* mCh HYG x *L*. *donovani* CTN PAC). Based on our previous results with *L*. *tropica*, we used stationary-phase promastigotes and LULO cells, and the addition of selection antibiotics was carried out on day 3 after mixing. Unfortunately, no hybrids were obtained under these conditions. Therefore, we decided to modify the promastigote phase of growth and use exponential-phase promastigotes in addition to stationary-phase promastigotes in the absence of LULO cells in an attempt to increase the probability of success, since as previously reported, mating-competent forms of *Leishmania* can also occur in axenic conditions [27]. Despite these modifications, we could not obtain intraclonal hybrids between these two *Leishmania* species. These results confirm the better capacity of *L*. *tropica* to generate mating-competent forms compared to *L*. *major* [45] and, as shown in this work, to *L*. *donovani* as well.

### Formation and characterization of interspecies hybrid strains *in vitro*

**Fig 3:**
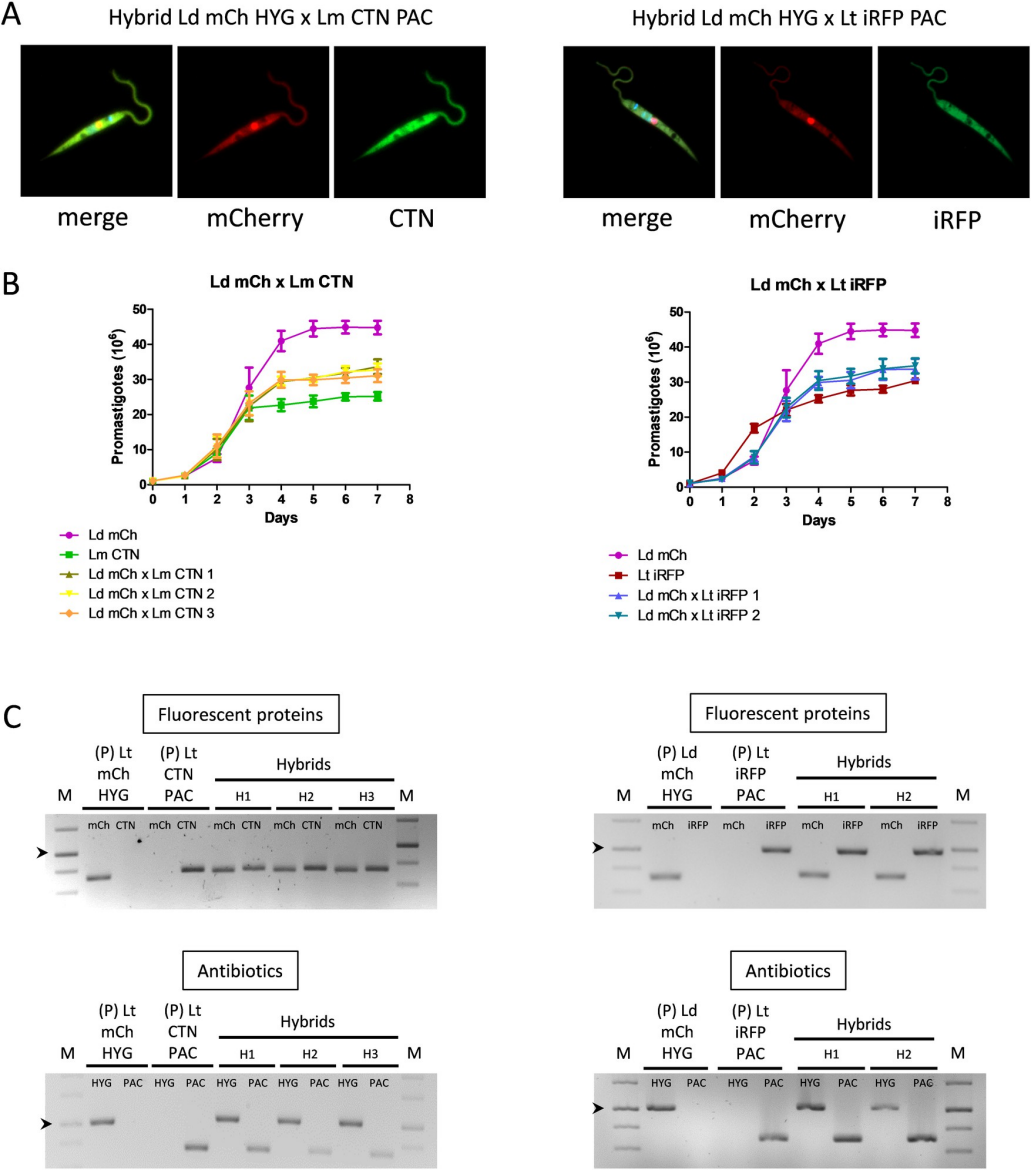
Characterization of interspecific hybrids. (A, left panel) Representative merged images of confocal microscopy and fluorescence emission in the mCh (561 nm) and CTN (488 nm) channels of one hybrid clone obtained after the outcrossing of *L*. *donovani* mCh HYG x *L*. *major* CTN PAC (Ld mCH HYG x Lm CTN PAC. (A, right panel) Representative merged images of confocal microscopy and fluorescence emission in the mCh (561 nm) and iRFP (640 nm) channels of one hybrid clone obtained after the outcrossing of *L*. *donovani* mCh HYG x *L*. *tropica* iRFP PAC (Ld mCH HYG x Lt iRFP PAC. (B, left panel) Growth curves (number of promastigotes in culture during 7 days) of the parental strains *L*. *donovani* mCh HYG (Ld mCh) and *L*. *major* CTN PAC (Lm CTN) and the interspecies hybrids Ld mCh x Lm CTN 1, 2 and 3. (B, right panel) Growth curves (number of promastigotes in culture during 7 days) of the parental strains *L*. *donovani* mCh HYG (Ld mCh) and *L*. *tropica* iRFP PAC (Lt iRFP), and the interspecies hybrids Ld mCh x Lt iRFP 1 and 2. (C, left panel) Agarose gels of the electrophoresis of the PCR products obtained after the amplification of the genes encoding fluorescent proteins (mCh and CTN) and antibiotic resistance (HYG or PAC) in the parental strains (P) and in the three interspecies hybrids (H) obtained after the outcrossing of *L*. *donovani* mCh HYG x *L*. *major* CTN PAC. (C, right panel) Agarose gels of the electrophoresis of the PCR products obtained after the amplification of the genes encoding fluorescent proteins (mCh and iRFP) and antibiotic resistance (HYG or PAC) in the parental strains (P) and in the two interspecies hybrids (H) resulting from the outcrossing of *L*. *donovani* mCh HYG x *L*. *tropica* iRFP PAC. The arrowheads indicate the 1000 bp band in the DNA molecular weight marker (M).

Interspecies hybrid formation was also attempted between *L*. *donovani* x *L*. *major*, *L*. *donovani* x *L*. *tropica* and *L*. *major* x *L*. *tropica*, using either exponential or stationary-phase promastigotes and in the presence and absence of LULO cells. Two different experiments were performed and addition of antibiotics was carried out on day 3 after mixing. As shown in Table 4, three hybrids (0.78% success) were obtained between stationary-phase promastigotes of *L*. *donovani* mCh HYG x *L*. *major* CTN PAC, which have yellow color (using red and green channels for visualization) (Fig 3A left panel) and are resistant to both hygromycin and puromycin. On the other hand, two hybrids (0.52% success) were formed in the absence of LULO cells between exponential-phase promastigotes of *L*. *donovani* mCh HYG x *L*. *tropica* iRFP PAC, which have yellow color (using red and far red channels for visualization) (Fig 3A right panel) and are resistant to both antibiotics. No hybrid progeny was obtained after crossing *L*. *tropica* x *L*. *major*, which can be related to the low success rate observed for the *in vitro* mating. Indeed, interspecies crosses occurred with a low success rate, and regardless the promastigote type stage (exponential or stationary phase) or the presence of an insect cellular feed layer, which confirms that LULO cells are not strictly required for *in vitro* formation of hybrids.

**Table 4:** Interspecies crossings performed with *L*. *donovani*, *L*. *major* and *L*. *tropica*, and the success rate (percentage of hybrids obtained) using different mating parental lines, different promastigote stages and in the presence and absence of LULO cells.

Crossing experiments	*L*. *tropica* iRFP PAC x *L*. *major* mCh HYG	*L*. *donovani* mCh HYG x *L*. *tropica* iRFP PAC	*L*. *donovani* mCh HYG x *L*. *major* CTN PAC
Exponential-phase promastigotes	Exp1: 0% (0/384)	**Exp 1: 0.52% (2/384)**	Exp 1: 0% (0/384)
	Exp 2: 0% (0/384)	Exp 2: 0% (0/384)	Exp 2: 0% (0/384)
Exponential-phase promastigotes+ LULO cells	Exp 1: 0% (0/384)	Exp 1: 0% (0/384)	Exp 1: 0% (0/384)
	Exp 2: 0% (0/384)	Exp 2: 0% (0/384)	Exp 2: 0% (0/384)
Stationary-phase promastigotes	Exp 1: 0% (0/384)	Exp 1: 0% (0/384)	Exp 1: 0% (0/384)
	Exp 2: 0% (0/384)	Exp 2: 0% (0/384)	Exp 2: 0% (0/384)
Stationary-phase promastigotes + LULO cells	Exp 1: 0% (0/384)	Exp 1: 0% (0/384)	Exp 1: 0% (0/384)
	Exp 2: 0% (0/384)	Exp 2: 0% (0/384)	**Exp 2: 0.78% (3/384)**

*L*. *donovani* mCh HYG x *L*. *major* CTN PAC and *L*. *donovani* mCh HYG x L. *tropica* iRFP PAC hybrids showed a growth profile in between their parental strains; the highest parasite number being provided by the parental *L*. *donovani* mCh HYG and the lowest parasite number being provided by the other parental strains (i.e. *L*. *major* CTN PAC and *L*. *tropica* iRFP PAC) (Fig 3B). Inheritance of the parental antibiotic resistance and reporter genes in the hybrid filial strains was confirmed by PCR amplification using the primers listed in Table 1. Fig 3C shows agarose gels of the amplification products in the parental and hybrid strains, thus confirming inheritance of the above-mentioned genes.

To the best of our knowledge, this is the first report describing *in vitro* interspecies outcrossing in *Leishmania*, and in this specific case, between species with different tropism; i.e. visceral (*L*. *donovani*) and cutaneous (*L*. *tropica* and *L*. *major*).

### Ploidy profile of hybrids

Once the hybrids were identified phenotypically, namely by their dual antibiotic resistance and mixed color detected by confocal microscopy, the ploidy of both the parental and filial generations was determined by flow cytometry in order to assess whether reductive or additive chromosome processes might have occurred after mating.

**Fig 4:**
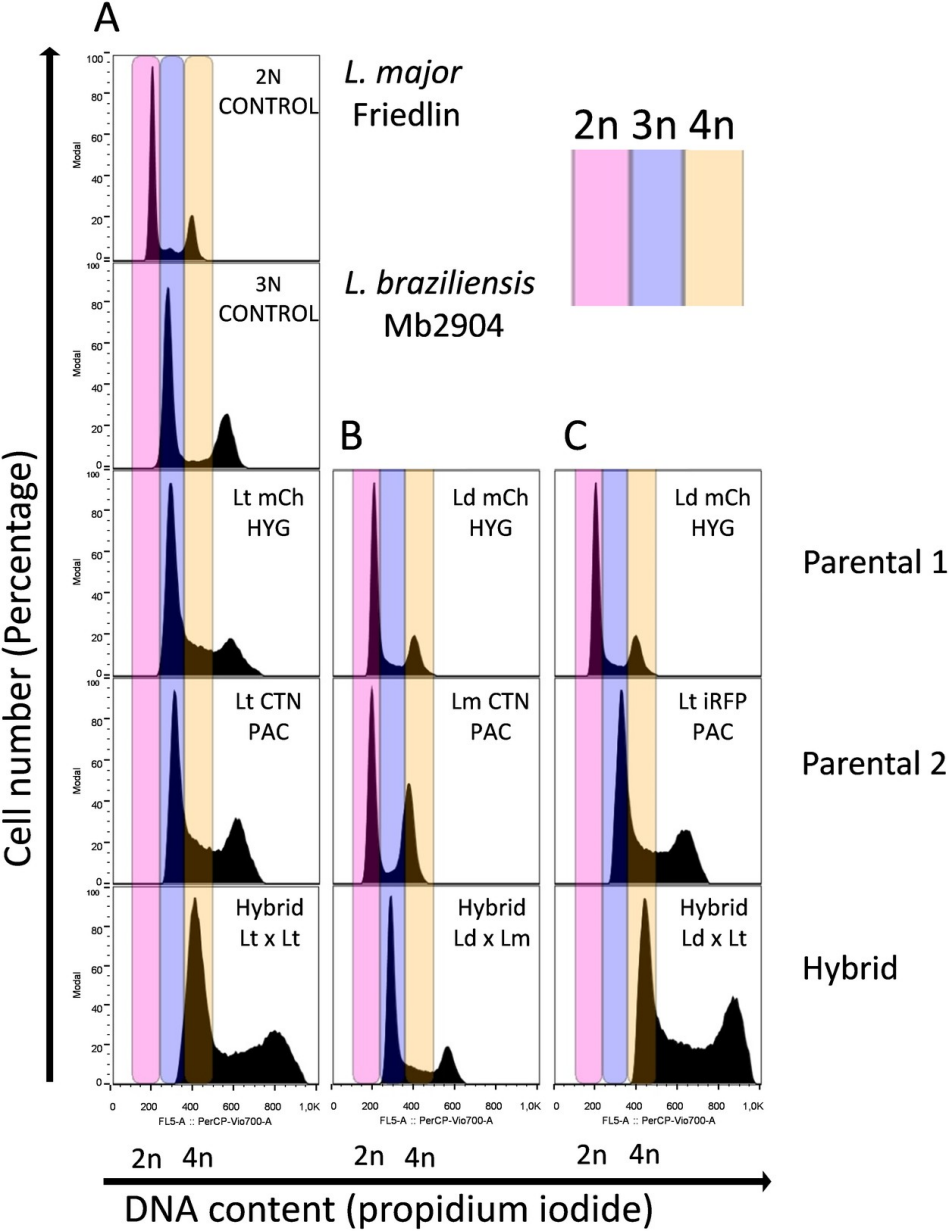
Analysis of the ploidy profile. (A) Representative chart of flow cytometry showing the ploidy of *L*. *tropica* mCh HYG (Lt mCh HYG), *L*. *tropica* CTN PAC (Lt CTN PAC) and one hybrid of the intraclonal crossing (Hybrid 1 Lt x Lt). (B) Representative chart of flow cytometry showing the ploidy of *L*. *donovani* mCh HYG (Ld mCh HYG), *L*. *major* CTN PAC (Lm CTN PAC) and one hybrid of the interspecies crossing (Hybrid 1 Ld x Lm). (C) Representative chart of flow cytometry showing the ploidy of *L*. *donovani* mCh HYG (Ld mCh HYG), *L*. *tropica* iRFP PAC (Lt iRFP PAC) and one hybrid of the interspecies crossing (Hybrid 1 Ld x Lt). *L*. *major* Friedlin (ploidy = 2n) and *L*. *braziliensis* Mb2904 (ploidy = 3n) were used as ploidy controls (2N CONTROL and 3N CONTROL, respectively) in order to assess the ploidy of the rest of the parasites.

Fig 4A shows a representative chart of flow cytometry of the result of *L*. *tropica* mCh HYG x *L*. *tropica* CTN PAC crossing (only one hybrid is shown, since all the hybrids exhibited identical results). As indicated by flow cytometry, the ploidy of the parental *L*. *tropica* strain was 3n, whereas ploidy of the filial strains after *in vitro* mating was approximately 4n. Regarding interspecies hybrids, flow cytometry results indicated that the three hybrids obtained from *L*. *donovani* x *L*. *major* (both 2n) were 3n (Fig 4B), while the ploidy of the two hybrids obtained from the *L*. *donovani* x *L*. *tropica* offspring was 4n (Fig 4C).

These results indicate that either intraclonal or interspecies crossings give rise to additive chromosome events in the hybrid progeny. This phenomenon has been previously observed by other authors, who have obtained in their crosses a mixture of hybrids with variable ploidy, with the presence of 2n, 3n and even 4n hybrids from 2n parents, regardless of whether the hybrids were obtained in the sandfly or *in vitro* [23–27]. In our work, all hybrids having a DNA content of 4n resulted from at least one 3n parental strain (*L*. *tropica*). As far as we know, intraclonal or interespecies hybrids from 3n parentals have never been described before, either in sandflies or *in vitro*.

### Genotypic characterization of interspecies hybrids

**Fig 5:**
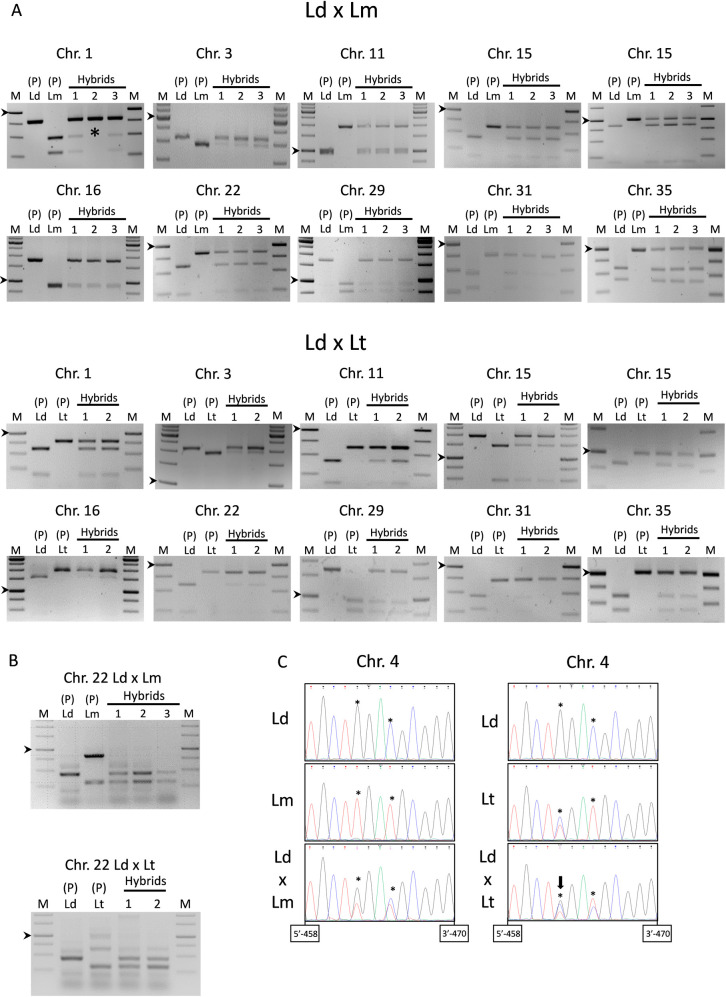
Characterization of the nuclear inheritance of the interspecies hybrids. (A) Agarose gels of the SNP-CAPs analysis showing the DNA products after PCR amplification and enzymatic digestion of the ten genes present in nine different chromosomes. The electrophoretic profile of the parental lines [P (Ld, Lm)] and three hybrids [Hybrids (1, 2, 3)] resulting from the outcrossing of *L*. *donovani* mCh HYG x *L*. *major* CTN PAC is shown in the top panel, whereas the electrophoretic profile of the parental lines [P (Ld, Lt)] and two hybrids [Hybrids (1, 2)] resulting from the outcrossing of *L*. *donovani* mCh HYG x *L*. *tropica* iRFP PAC is shown in the bottom panel. Note the absence of the *L*. *major* allele of the gene located on chromosome 1 in hybrid number two (indicated with an asterisk). (B) Agarose gels of the PCR amplification of the A2 gene in the parental lines [P (Ld, Lm)] and three hybrids [Hybrids (1, 2, 3)] resulting from the outcrossing of *L*. *donovani* mCh HYG x *L*. *major* CTN PAC (top panel), and in the parental lines [P (Ld, Lt)] and two hybrids [Hybrids (1, 2)] resulting from the outcrossing of *L*. *donovani* mCh HYG x *L*. *tropica* iRFP PAC (bottom panel). The arrowheads indicate the 1000 bp band in the DNA molecular weight marker (M). (C) Fluorograms showing the sequence of part of the actin gene and some SNPs (marked with an asterisk) in the parental strains *L*. *donovani* mCh HYG (Ld), *L*. *major* CTN PAC (Lm) and *L*. *tropica* iRFP PAC (Lt), and one representative hybrid of the outcrossing between *L*. *donovani* mCh HYG x *L*. *major* CTN PAC (Ld x Lm) and *L*. *donovani* mCh HYG x *L*. *tropica* iRFP PAC (Ld x Lt). Note the triple nucleotide peaks in the fluorograms of *L*. *donovani* x *L*. *tropica* hybrid (arrow in the SNP located at position 462 in the right panel).

Genotypic characterization of the interspecies hybrids was initially performed by SNP-CAPs in ten genes encoding the following proteins: calmodulin-binding protein, ABC transporter, Sec20, Rad9, cytoplasmic L-asparaginase i-like protein, paraflagellar rod protein, ribosomal protein L22p/L17e, fumarate hydratase, aquaglyceroporin 1, and 2-oxoisovalerate dehydrogenase beta subunit, which are present in different *Leishmania* chromosomes as indicated in Table 2. For this purpose, these genes were amplified with specific primers and cut with suitable restriction enzymes (see Table 2 for details). Amplification and restriction analysis allowed us to characterize the SNPs present in the progenitor strains and in the interspecies hybrids resulting from crosses of *L*. *donovani* mCh HYG x *L*. *major* CTN PAC and *L*. *donovani* mCh HYG x *L*. *tropica* iRFP PAC (Fig 5A).

After amplification and restriction analyses it can be observed that the bands corresponding to both progenitors are visible in the three hybrids of *L*. *donovani* x *L*. *major* outcrossing, which would correspond to a biparental inheritance produced by a genetic exchange after mating. As an exception we can highlight the absence of the *L*. *major* allele of the gene located on chromosome 1 in one of the hybrids (Fig 5A top panel) that can be due to a loss of heterozygosity and/or aneuploidy in *Leishmania*, as it has been previously reported [26]. Similar conclusions are raised from the crossing of *L*. *donovani* x *L*. *tropica* (Fig 5A bottom panel), where the biparental inheritance of the hybrids appears again represented by the bands corresponding to the enzymatic restriction of the amplified gene of both progenitors.

We also analysed the composition of A2 gene in the hybrids after mating. The interest of the product of A2 gene is because it has been involved in the organic tropism of visceralizing *Leishmania* strains including *L*. *donovani* [46]. In this species, the A2 gene is represented by a multicopy cluster (tandem-repeats) in chromosome 22 and encodes a family of A2 proteins with 10-amino acid repeated sequences. However, a single and truncated pseudogene is present in the species with cutaneous tropism, namely *L*. *major* and *L*. *tropica* [46,47]. For the amplification of this gene, we used the primers L2/R3 (Table 1), which were previously described by Garin et al. (2005) [46] and later used by Romano et al. (2014) [26]. As we can see in Fig 5B, the parental *L*. *donovani* strain shows a main band near 500 bp, whereas *L*. *major* and *L*. *tropica* show a main band over 250 bp, which correspond to the pattern amplified by these primers in these species [46,48]. The presence of the main amplification bands that are visible in both parental strains and in the hybrid clones from both crosses, further demonstrates biparental inheritance (Fig 5B).

Finally, nuclear inheritance was also demonstrated by sequencing the actin gene located on chromosome 4, which was amplified using primers TOX83 and TOX84 (Table 1). Sequence analysis (Fig 5C) of several regions confirmed the inheritance of the SNPs located at positions 462 and 465 of the actin gene (from the initial ATG codon) in the hybrids obtained from both interspecies outcrossings. The parental *L*. *tropica* strain turned out to be heterozygous for some SNPs. This led to the visualization of triple nucleotide peaks in the sequencing fluorograms of *L*. *donovani* x *L*. *tropica* hybrids (Fig 5C right panel), which inherited at least two copies of the parental *L*. *tropica* and at least one copy of the parental *L*. *donovani* actin gene.

**Fig 6:**
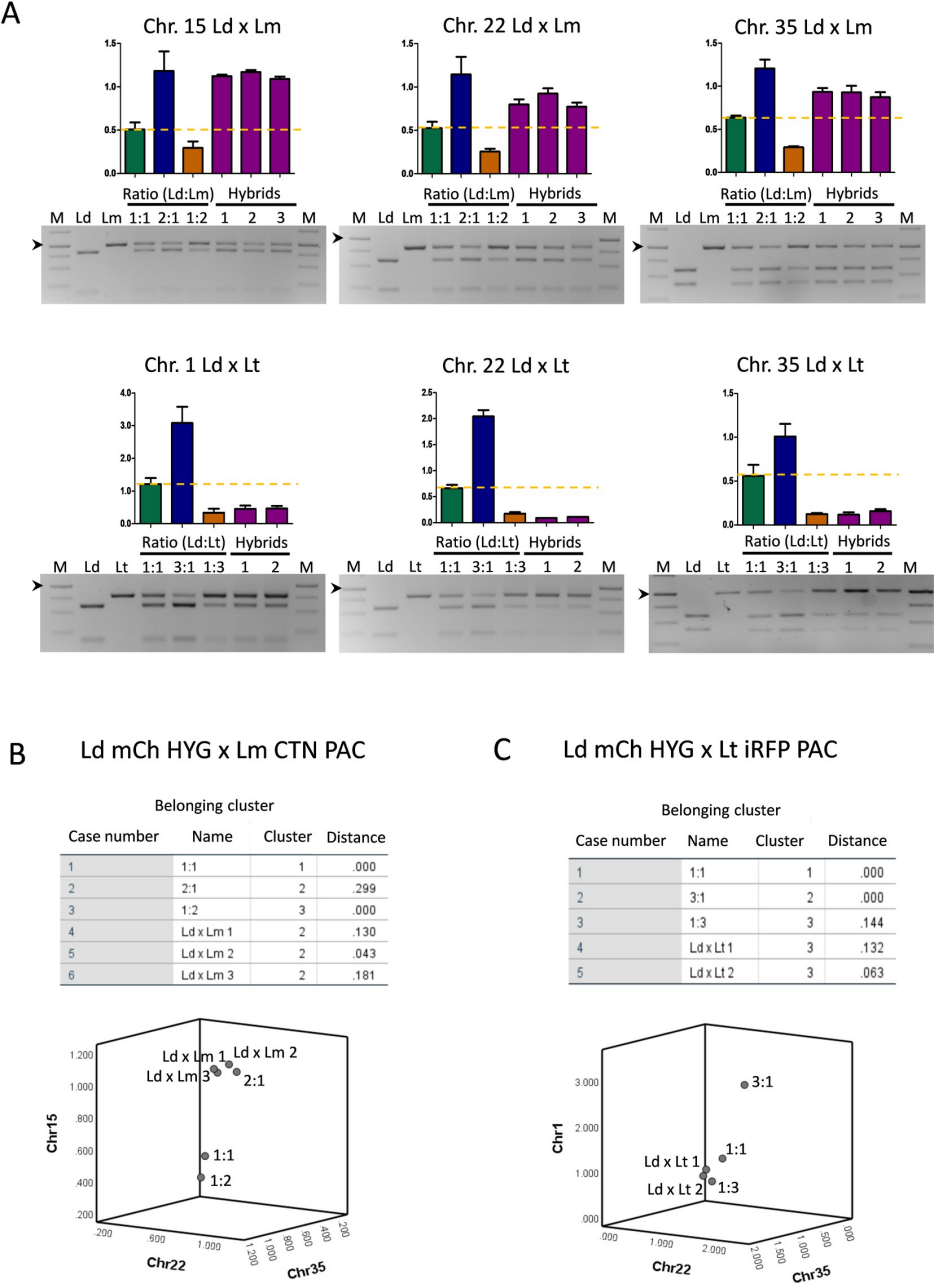
Characterization of the inheritance of extra chromosome sets in the interspecies hybrid clones. (A) Agarose gels of the SNP-CAPs analysis showing the DNA products after PCR amplification and enzymatic digestion of three loci present in chromosomes 2, 22 and 35. The top panel shows the electrophoretic profile of *L*. *donovani* mCh HYG (Ld), *L*. *major* CTN PAC (Lm) and three hybrids (Ld x Lm), and the ratios between the intensity of the upper band from the parental *L*. *donovani* and the uncut upper band from the other parental *L*. *major* in the corresponding hybrid clones. Simulated 3n were created by mixing parental DNA in 1:1, 1:2 and 2:1 ratio (*L*. *donovani*:*L*. *major*). The bottom panel shows the electrophoretic profile of *L*. *donovani* mCh HYG (Ld), *L*. *tropica* iRFP PAC (Lt) and two hybrids (Ld x Lt), and the ratios between the intensity of the upper band from the parental *L*. *donovani* and the uncut upper band from the other parental *L*. *tropica* in the corresponding hybrid clones. Simulated 4n were created by mixing parental DNA in 1:1, 1:3 and 3:1 ratio (*L*. *donovani*:*L*. *tropica*). The arrowhead indicates 1000 bp in the DNA molecular weight marker (M). (B, C) Statistical representation by k-means analysis in SPSS of the extrachromosome inheritance of *L*. *donovani* mCh HYG x *L*. *major* CTN PAC (Ld mCh HYG x Lm CTN PAC) hybrids, which are clustered together with ratio 2:1 (LdxLm) (B), and *L*. *donovani* mCh HYG x *L*. *tropica* iRFP PAC (Ld mCh HYG x Lt iRFP PAC) hybrids, the latter being clustered together with ratio 1:3 (LdxLt) (C).

The origin of the extra chromosome sets in the different hybrids was determined as indicated in the Materials and methods section. Fig 6A shows the SNP-CAPs digestion products for different genes encoding the following proteins: calmodulin-binding protein (chromosome 1), cytoplasmic L-asparaginase i-like protein (chromosome 15), ribosomal protein L22p/L17e (chromosome 22) and 2-oxoisovalerate dehydrogenase beta subunit (chromosome 35) (see Table 2 for details), and the ratios between the intensity of the upper band from the parental *L*. *donovani* and the uncut band from the other parental (*L*. *major* or *L*. *tropica*) in the corresponding hybrid clones. Results indicated that the hybrids obtained from *L*. *donovani* x *L*. *major* crosses, inherited the extra trisomic chromosomes 15, 22 and 35 from *L*. *donovani* (Fig 6A top panel). On the contrary, the hybrids obtained from *L*. *donovani* x *L*. *tropica* crossing seem to have inherited the extra chromosomes 1, 22 and 35 from *L*. *tropica* (Fig 6A botton panel). This is consistent with the results represented in Fig 5C, which showed that at least two copies of the actin gene would have been inherited from *L*. *tropica*. Statistical analysis of the ratios showed that *L*. *donovani* x *L*. *major* hybrids are grouped within the category 2:1 (*L*. *donovani*:*L*. *major*) (Fig 6B), whereas *L*. *donovani* x *L*. *tropica* hybrids are grouped within the category 1:3 (*L*. *donovani*:*L*. *tropica*) (Fig 6C), thus confirming the inheritance of extrachromosome sets from *L*. *donovani*, in the case of *L*. *donovani* x *L*. *major* hybrids (3n), or *L*. *tropica* in the case of *L*. *donovani* x *L*. *tropica* hybrids (4n).

The differences in ratios for each locus may reflect mosaic aneuploidy in the progeny. This process has been demonstrated in several *Leishmania* species [49], and has been suggested to represent a unique source of adaptability for the parasites to new environment [50].

### Extranuclear DNA inheritance of interspecies hybrid clones

**Fig 7:**
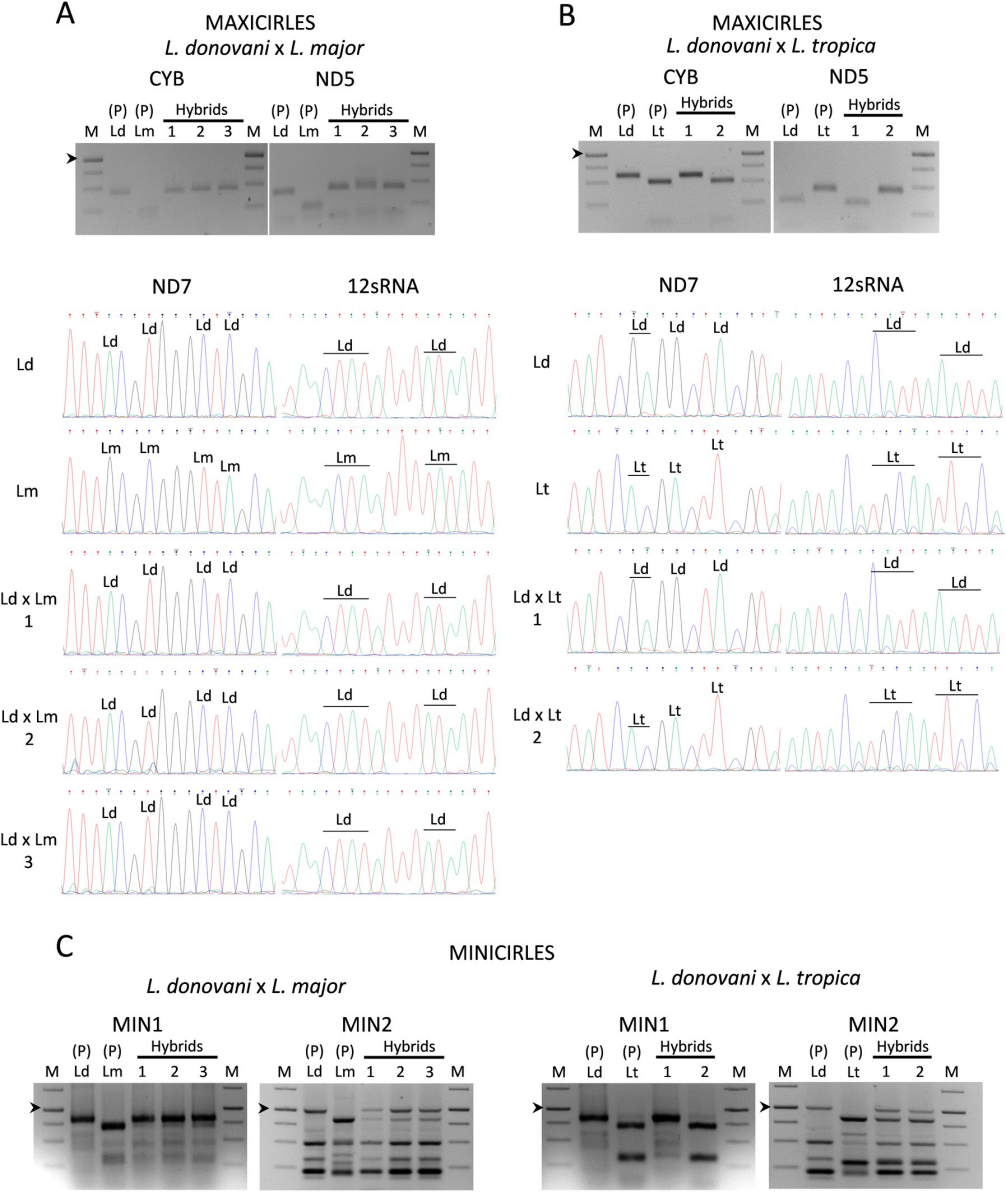
Characterization of the inheritance of kDNA in the interspecies hybrid clones. (A, B, top panels) Agarose gels of the SNP-CAPs analysis showing the DNA products after PCR amplification and enzymatic digestion of *Cyb* and *ND5* genes. The electrophoretic profile of the parental lines [P (Ld, Lm)] and three hybrids [Hybrids (1, 2, 3)] resulting from the outcrossing of *L*. *donovani* mCh HYG (Ld) x *L*. *major* CTN PAC (Lm) (A), or the parental lines [P (Ld, Lt)] and two hybrids [Hybrids (1, 2)] resulting from the outcrossing *L*. *donovani* mCh HYG (Ld) x *L*. *tropica* iRFP PAC (Lt) (B), are shown. (A, B bottom panels) Fluorograms showing the sequence of part of the *ND7* and *12 sRNA* genes with some SNPs, which have been indicated as Ld (*L*. *donovani*) and Lm (*L*. *major*) in the parental strains *L*. *donovani* mCh HYG (Ld), *L*. *major* CTN PAC (Lm) and in their clonal hybrids (LdxLm) 1, 2 and 3 (A), or as Ld (*L*. *donovani*) and Lt (*L*. *tropica*) in the parental strains *L*. *donovani* mCh HYG (Ld), *L*. *tropica* iRFP PAC (Lt) and in their clonal hybrids (LdxLt) 1 and 2 (B). (C) Agarose gels showing the band profile after amplification of a conserved region (MIN1) or a variable region (MIN2) of minicircles in the parental lines [P (Ld, Lm)] and three hybrids [Hybrids (1, 2, 3)] resulting from the outcrossing of *L*. *donovani* mCh HYG (Ld) x *L*. *major* CTN PAC (Lm) (left panel), or in the parental lines [P (Ld, Lt)] and two hybrids [Hybrids (1, 2)] resulting from the outcrossing *L*. *donovani* mCh HYG (Ld) x *L*. *tropica* iRFP PAC (Lt) (right panel). The arrowhead indicates 1000 bp in the DNA molecular weight marker (M).

The inheritance of maxi and minicircles of extrachromosomal kDNA in the interspecies hybrids was also analysed. For the study of the inheritance of maxicircles, four genes (*Cyb*, *ND5*, *ND7* and *12sRNA*) were analysed. Inheritance of *Cyb* and *ND5* was assessed by SNP-CAPs (see Table 2 for details). As seen in the top panel of Fig 7A, interspecies hybrid clones of *L*. *donovani* mCh HYG x *L*. *major* CTN PAC only exhibit the restriction pattern of the parental *L*. *donovani* strain for these two genes. In the case of the *L*. *donovani* mCh HYG x *L*. *tropica* iRFP PAC interspecies outcrossing, one of the hybrid clones showed a restriction pattern for *Cyb* and *ND5* similar to that observed for *L*. *donovani*, whereas the other hybrid clone showed a behavior similar to the other parental (i.e. *L*. *tropica*) (Fig 7B top panel). In order to confirm this phenomenon, the analysis of the inheritance of *ND7* and *12sRNA* was performed by sequencing these genes after amplification with the primers TOX97, TOX98 and TOX101, TOX102 (Table 1). Fluorograms indicated that in the three hybrid clones *L*. *donovani* mCh HYG x *L*. *major* CTN PAC, the SNPs were inherited from the parental *L*. *donovani* strain (Fig 7A bottom panel), whereas one of the hybrid clones of the *L*. *donovani* mCh HYG x *L*. *tropica* iRFP PAC outcrossing inherited the SNPs from *L*. *donovani* and the other hybrid clone inherited the SNPs from *L*. *tropica* (Fig 7B bottom panel). These results indicate that inheritance of maxicircles is uniparental or alternatively, that genetic information contained in maxicircles from one of the parental strains can be lost by genetic drift, as it has been previously suggested in *T*. *brucei* [31].

For the study of the inheritance of minicircles, PCR experiments were conducted with primers TOX73 and TOX 74, which amplify near 780 bp from a conserved region (MIN1) in minicircle kDNA in *Leishmania* spp. [39] (Table 1), and with primers TOX75 and TOX76 (Table 1), the latter amplifying a variable region of minicircles (MIN2) and generating multi-sized products [40]. As shown in Fig 7C, hybrids from either *L*. *donovani* mCh HYG x *L*. *major* CTN PAC or *L*. *donovani* mCh HYG x *L*. *tropica* iRFP PAC exhibited an amplification pattern of MIN1 that included bands from both parental strains, although with different intensity. In the case of *L*. *donovani* mCh HYG x *L*. *major* CTN PAC, the most intense band in the three hybrid clones corresponds to that inherited from *L*. *donovani*, which correlates well with the results obtained with the inheritance of maxicircles.

The pattern for MIN2 in hybrids from either *L*. *donovani* mCh HYG x *L*. *major* CTN PAC or *L*. *donovani* mCh HYG x *L*. *tropica* iRFP PAC, provided a more precise picture of the presence of bands from both parental strains, thereby suggesting the biparental inheritance of minicircles.

### Infectivity of intraclonal and interspecies hybrids

**Fig 8:**
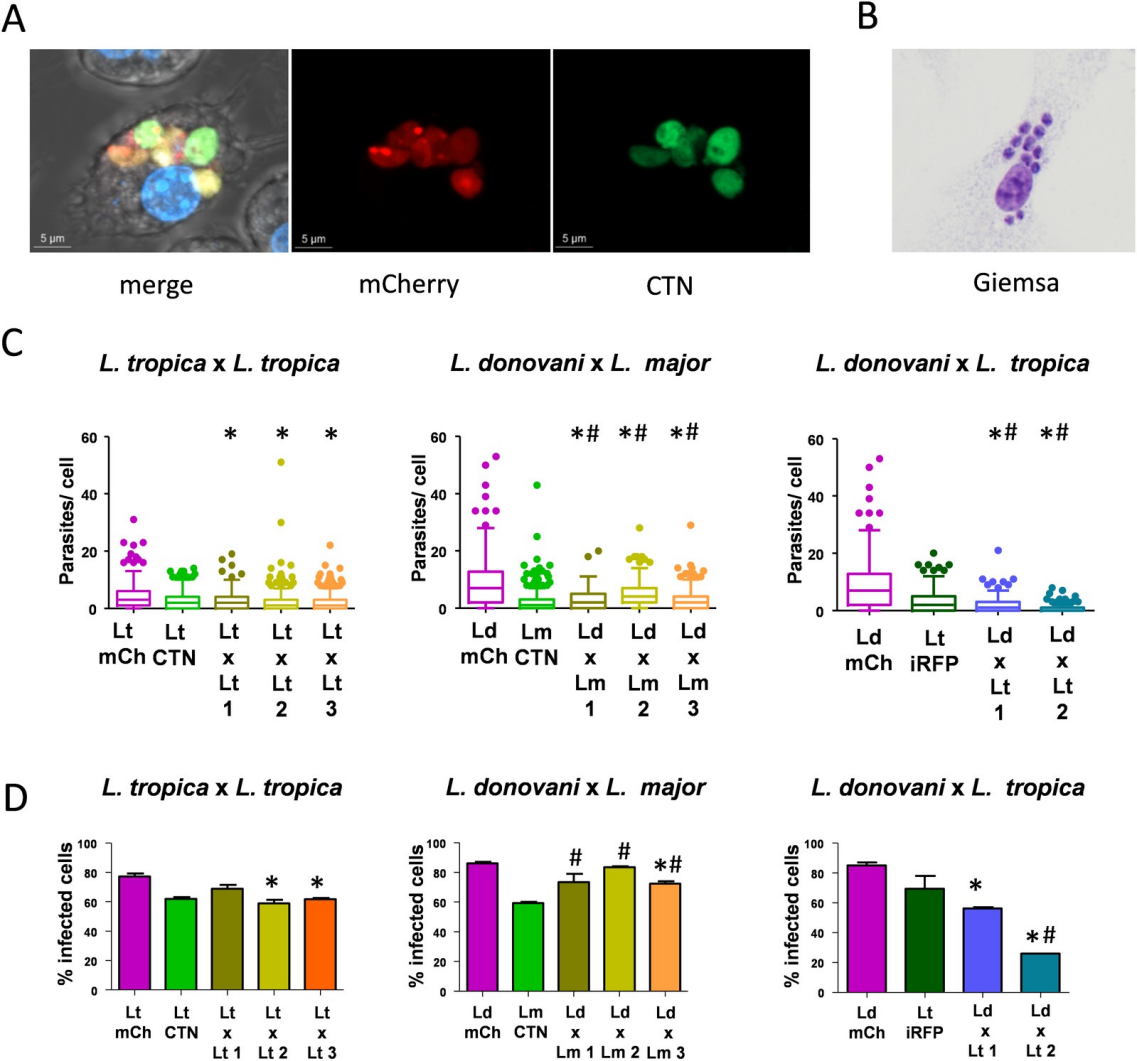
Infectivity of intraclonal and interspecies hybrids. (A) Representative image of confocal microscopy of a RAW cell infected with one hybrid of *L*. *donovani* mCh HYG x *L*. *major* CTN PAC. Merge and fluorescent pictures in the mCh (561 nm) and CTN (488 nm) channels are shown. (B) Representative image of BMMs infected with amastigotes of one hybrid of L. *donovani* mCh HYG x *L*. *major* CTN PAC and stained with Giemsa. (C) Box plots representing the number of amastigotes present within each BMM after *in vitro* infection withamastigotes of *L*. *tropica* mCh HYG (Lt mCh), *L*. *tropica* CTN PAC (Lt CTN), three intraclonal hybrids *L*. *tropica* mCh HYG x *L*. *tropica* CTN PAC (Lt x Lt 1, 2 and 3), *L*. *donovani* mCh HYG (Ld mCh), *L*. *major* CTN PAC (Lm CTN) three interspecies hybrids *L*. *donovani* mCh HYG x *L*. *major* CTN PAC (Ld x Lm 1, 2 and 3), *L*. *tropica* iRFP PAC (Lt iRFP), and two interspecies hybrids *L*. *donovani* mCh HYG x *L*. *tropica* iRFP PAC (Ld x Lt 1 and 2). (D) Bar graphs representing the percentage of infected BMMs after *in vitro* infection withamastigotes of *L*. *tropica* mCh HYG (Lt mCh), *L*. *tropica* CTN PAC (Lt CTN), three intraclonal hybrids *L*. *tropica* mCh HYG x *L*. *tropica* CTN PAC (Lt x Lt 1, 2 and 3), *L*. *donovani* mCh HYG (Ld mCh), *L*. *major* CTN PAC (Lm CTN) three interspecies hybrids *L*. *donovani* mCh HYG x *L*. *major* CTN PAC (Ld x Lm 1, 2 and 3), *L*. *tropica* iRFP PAC (Lt iRFP), and two interspecies hybrids *L*. *donovani* mCh HYG x *L*. *tropica* iRFP PAC (Ld x Lt 1 and 2). Differences were considered as significant when p<0,05 and in a Kruskal-Wallist test (C) or in a one-way ANOVA analysis (D) (represented with an asterisk regarding the first parental line and with a hash regarding the second parental line).

*In vitro* infection of hybrid clones obtained from different mating experiments was evaluated in BMMs. The objective of this experiment was the phenotypic comparison using amastigotes from the hybrids and the parental species under the same infection conditions. The ability of hybrids to infect macrophages was first checked in RAW cells by confocal microscopy (Fig 8A) because BMMs show high autofluorescence that interferes with visualization of amastigotes. Once the ability of these parasites to infect macrophages was confirmed *in vitro*, the infectivity of amastigotes was tested in BMMs derived from Balb/c mice by microscopy analysis after Giemsa staining (Fig 8B). The number of parasites infecting each BMMs was determined after the analysis of 100 BMMs in triplicate and represented for each hybrid (Fig 8C). Results indicate that amastigotes of all the intraclonal hybrids showed a reduction in their infection ability regarding *L*. *tropica* mCh HYG. Interspecies hybrids *L*. *donovani* mCh HYG x *L*. *major* CTN PAC showed infectivity values between both parental strains. However, amastigotes of interspecies hybrids *L*. *donovani* mCh HYG x *L*. *tropica* iRFP PAC exhibited reduced infectivity values in comparison with both parental strains (Fig 8C). Regarding the number of macrophages that hybrid and parental parasites were able to infect (Fig 8D), intraclonal hybrids *L*. *tropica* mCh HYG x *L*. *tropica* CTN PAC 2 and 3 showed a significant decrease regarding the parental *L*. *tropica* mCh HYG. Infection with amastigotesof the interspecies hybrids *L*. *donovani* mCh HYG x *L*. *major* CTN PAC did not show significant differences regarding *L*. *donovani* mCh HYG, although a significant increase in the percentage of infected BMMs was observed regarding the other parental strain *L*. *major* CTN PAC. The exception was hybrid 3, which exhibited a significant decrease in the percentage of infected BMMs regarding *L*. *donovani* mCh HYG. Infection with either amastigotes of the interspecies hybrids *L*. *donovani* mCh HYG x *L*. *tropica* iRFP PAC, in general, provided a reduction in the percentage of infected BMMs regarding both parental strains, this decrease being more patent in the case of interspecies hybrid 2 (Fig 8D).

Hybrid promastigotes were also able to infect mice, and amastigotes were recovered from the spleens of infected mice. The recovered parasites maintained double antibiotic resistance, thus confirming stability of the hybrids upon *in vivo* infection.

In conclusion, we have demonstrated in this work that outcrossing in *Leishmania* can also occur under *in vitro* conditions, even between species of different tropism and even if a parental strain contains a ploidy of 3n. In addition, the resultant 4n hybrids are stable after *in vivo* infection.

With the data obtained, the mechanism underlying sexual reproduction in the crosses could be related with meiosis in which the hybrids contain one allele of each parent for the markers analysed. On the other hand, polyploidy hybrids are frequently formed after outcrossings of Trypanosoma and Leishmania species. However, it has been reported that most of those crosses correspond to a meiotic cycle [7,9,51]. This could be explained by the existence of a tetraploid meiotic cycle in which one of the parental nucleus does not undergo meiosis after cell fusion [45], or alternatively, by the fusion of meiotic intermediates with gametes [8].

These results contribute to the understanding of sexual reproduction in this trypanosomatid and suggest that mechanisms of virulence and drug resistance can be acquired by *Leishmania* through this process.

## Supporting information

S1 Fig Schematic representation of pLEXSY-CTN-PAC. (TIF)
