# Supplementary figures and images for: Axenic interspecies and intraclonal hybrid formation in Leishmania: Successful crossings between visceral and cutaneous strains
Source: PLoS Negl Trop Dis. 2022 Feb 9;16(2):e0010170. doi: 10.1371/journal.pntd.0010170 (PMC8827483; doi:10.1371/journal.pntd.0010170)

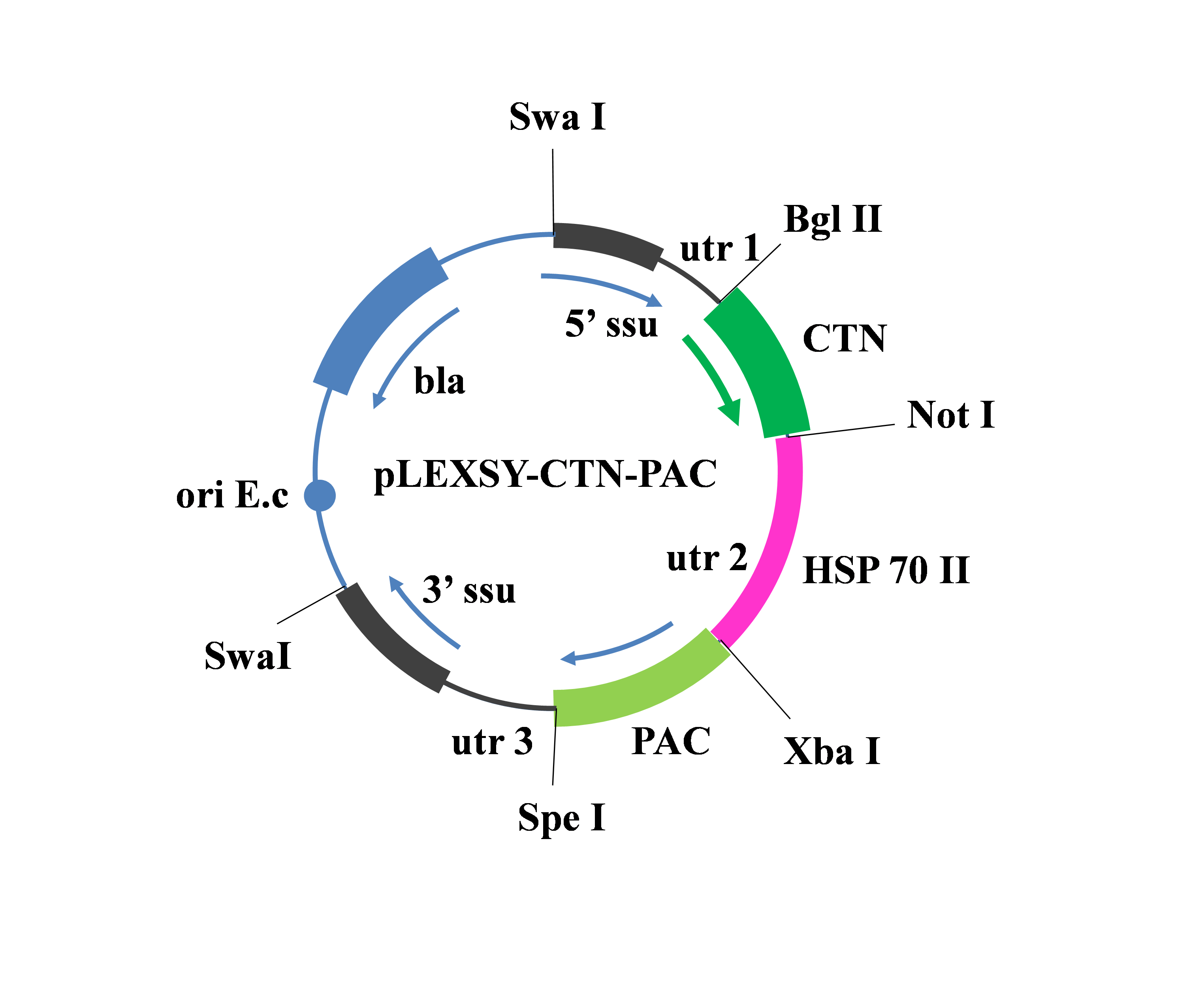

Supplement: S1 Fig — (TIF) [file pntd.0010170.s001.tif]
